# Proteomic Analysis of *Listeria monocytogenes* Subjected to Pulsed Magnetic Field

**DOI:** 10.3390/foods14111871

**Published:** 2025-05-24

**Authors:** Di Chen, Jingya Qian, Shuhao Huo, Feng Wang, Haile Ma, Shan Liu

**Affiliations:** 1 School of Food and Biological Engineering, Jiangsu University, 301 Xuefu Road, Zhenjiang 212013, China; 2212218011@stmail.ujs.edu.cn (D.C.); huoshuhao@yeah.net (S.H.); fengwang@ujs.edu.cn (F.W.); mhl@ujs.edu.cn (H.M.); 2 Institute of Food Physical Processing Engineering, Jiangsu University, 301 Xuefu Road, Zhenjiang 212013, China; 3 College of Engineering, China Agricultural University, Beijing 100083, China; liu610shan@cau.edu.cn

**Keywords:** pulsed magnetic field (PMF), *Listeria monocytogenes*, inactivation, mechanism, proteomic analysis

## Abstract

As one of the non-thermal technologies, the pulsed magnetic field (PMF) has increasingly attracted attention for its application in food microbial inactivation. In this study, a proteomic analysis was conducted to elucidate the molecular mechanism underlying the inactivation of *Listeria monocytogenes (L. monocytogenes)* by a PMF. A total of 79 proteins, comprising 65 upregulated and 14 downregulated proteins, were successfully identified as differentially expressed proteins (DEPs, >1.2-fold or <0.83-fold, *p*-value < 0.05) in *Listeria monocytogenes* exposed to a PMF at 8 T with 20 pulses. Gene Ontology (GO) and Kyoto Encyclopedia of Genes and Genomes (KEGG) pathway analyses revealed that PMF exposure significantly impacted nutrient transport, the composition of cytoplasmic and intracellular substances, and various metabolic processes in *L. monocytogenes*, such as carbohydrate metabolism, amino acid metabolism, and nicotinate and nicotinamide metabolism. The disruption of cellular functions and metabolic pathways may contribute to the death of *L. monocytogenes* under PMF treatment. These findings provide valuable insights and serve as a reference for further investigations into the inactivation mechanisms induced by PMFs.

## 1. Introduction

The growing demand for high-quality or minimally processed food is driving the development of innovative technologies in food processing. Non-thermal technologies do not generate high temperatures during food processing and have relatively short processing times. Non-thermal processing results in better retention of nutrients in foods than that in traditional thermal processing [1]. Non-thermal technologies, such as high pressure [2], ultrasound [3], and pulsed electric field [4], are capable of inactivating microorganisms while inducing minimal changes to the sensory and nutritional properties of food. The PMF has emerged as one of the non-thermal technologies and can effectively reduce microbial loads in liquid foods, such as fruit juices, under ambient pressure and temperature conditions [5]. Several studies have highlighted its efficacy in eliminating pathogens like *Escherichia coli* O157:H7 [6] and *Listeria grayi* [7] in liquid matrices, including juice and buffer solutions. The study by Ragupathi et al. showed that a PMF effectively inhibited the growth of tomato pathogenic bacteria [8]. Compared with conventional heat treatment and chemical processing methods, PMF technology shows advantages such as reduced process duration, environmentally friendly operation without additional damage, and no chemical residues [7].

However, the inactivation mechanisms of the PMF toward the microorganisms have not been fully elucidated. Cell membranes and walls are the main targets of the PMF. The PMF induced the formation of pores, changes in permeability, and structural disruption of the cell membrane, resulting in the leakage of cellular components [5]. Furthermore, changes in mobility, carbohydrate metabolism, energy metabolism, amino acid metabolism, phosphorylation and dephosphorylation, membrane properties, quorum sensing, two-component regulatory systems, and ATP-binding cassette (ABC) transporters were related to cell death after PMF treatment [9].

*L. monocytogenes* is one of the major foodborne pathogens that can cause a rare and severe disease called listeriosis [10]. In recent years, there has been a notable rise in *L. monocytogenes* outbreaks across Europe and the United States. *L. monocytogenes* was identified as the primary cause of zoonosis-related deaths in 37 European countries, accounting for 52% of deaths associated with outbreaks in the United States. Dairy products, such as soft cheese [11] and raw fluid milk [12]; ready-to-eat foods, such as smoked salmon [13] and cooked meats [14]; and fresh produce, such as apple [15] and lettuce [16], have been closely associated with *L. monocytogenes* contamination. The pathogenic microorganism *L. monocytogenes*, a significant concern in the food industry, can be transmitted to humans, posing a substantial risk to immunocompromised individuals, pregnant women, newborns, and the elderly [17]. This pathogen not only poses a significant threat to public health but also presents substantial challenges to food safety management, necessitating urgent attention from public health authorities globally [18]. Therefore, the inactivation of *L. monocytogenes* in both processed and fresh foods is essential for ensuring food safety.

The efficacy of non-thermal approaches in reducing *L. monocytogenes* has been documented in several studies. High-Pressure Processing (HPP) treatments, particularly at 600 MPa and to a lesser extent at 500 MPa, are effective to eliminate *L. monocytogenes* in fresh cheeses [19], and a reduction of 6.2 log CFU/g was achieved when simulated meat was treated with HPP at 600 MPa for 5 min. Pulsed electric field (PEF) treatment resulted in *L. monocytogenes* population reductions ranging from 18.98% to 43.64% as the field strength was increased from 15 to 30 kV/cm in milk [20]. Power was the predominant factor influencing *L. monocytogenes* inactivation in almond milk using ultrasound, and a 1 log CFU/mL reduction in viable count was achieved when the power was set to 80% for 8 min [21].

Proteomic approaches have been employed to investigate bacterial cellular responses under specific conditions [22,23]. By detecting and identifying proteins and analyzing protein–protein interactions, biological events can be elucidated. Moreover, insights into bacterial metabolic pathways can be gained, and this knowledge can facilitate a deeper understanding of the mechanisms underlying cellular responses to stimuli. Proteomics analysis has been effectively utilized to investigate global changes in protein expression in biological organisms under diverse environmental conditions [24]. Therefore, proteomic approaches have been widely utilized as a primary tool to investigate microbial responses to antibacterial agents [25,26]. In this study, a tandem mass tag (TMT)-based quantitative proteomic analysis was performed to investigate the proteomic changes of *L. monocytogenes* exposed to a PMF, thereby elucidating the molecular mechanisms underlying PMF-induced inactivation.

## 2. Materials and Methods

### 2.1. L. monocytogenes Sample Preparation

*L. monocytogenes* (ATCC 19111, American Tissue Culture Collection, ATCC) was stored at −20 °C in a 10% glycerol solution. Cells were subcultured in brain heart infusion broth (BHI, Shanghai Sinopharm Group Chemical Reagent Co., Ltd., Shanghai, China) at 37 °C for 24 h. A single colony was selected and inoculated into 50 mL of BHI broth, incubated at 37 °C for 24 h. Subsequently, 1 mL of the culture was transferred to 50 mL of fresh BHI broth and incubated in a shaking incubator at 37 °C with a shaking speed of 180 rpm for 4 h to reach the exponential growth phase. The cells were harvested by centrifugation at 6000× *g* for 5 min at 4 °C and subsequently resuspended in 4 mL of phosphate-buffered saline (PBS, pH 7.2).

### 2.2. PMF Treatment

The pulsed magnetic field device (TSK-H15300, Tingjin Magnetic Components, Nanjing, China) consisted of a magnetic field generator and a treatment chamber. A magnetic field generator was employed to produce a pulsed magnetic field by charging and discharging current within the treatment chamber. The treatment chamber consisted of a coil measuring 0.5 m in length and 5 cm in diameter. The intensity of PMF was quantified using a teslameter (LZ-610H, Hunan Linkjoin Technology, Hunan, China). The *L. monocytogenes* samples were exposed to PMF intensities ranging from 2 to 8 Tesla (T) and pulse numbers varying between 10 and 50. The duration of a single pulse was maintained at 0.3 ms throughout the experimental treatments. Circulating cooling water was employed to maintain the treatment temperature at room temperature throughout the process. The samples that were not exposed to PMF treatment served as the control group. All the experiments were conducted in triplicate.

### 2.3. Determination of L. monocytogenes Inactivation

An aliquot of 0.1 mL of the sample was subjected to a 10-fold serial dilution with 0.9 mL PBS, and 0.1 mL of appropriately diluted sample an appropriate dilution was evenly spread onto the BHI agar plates. Each dilution was performed in duplicate, and the plates were incubated at 37 °C for 24 h before the colonies were counted. The detection limit of colony count is defined as the lowest sample concentration at which microorganisms can be reliably detected, corresponding to a colony count of 20 CFU per plate. The survival fraction (N/N_0_) was calculated to represent the inactivation efficiency, where N_0_ and N were colony-forming units (CFUs) of *L. monocytogenes* before and after PMF treatment, respectively.

### 2.4. Protein Extraction and Quantitation

*L. monocytogenes* samples before and after PMF treatment were lysed with 300 µL lysis buffer supplemented with 1 mM PMSF. The samples were sonicated at 80 W with a pulse cycle of 1 s on/1 s off applied to the samples for 3 min. After the sonication, the samples were centrifuged at 15,000× *g* for 15 min to remove insoluble particles. To ensure consistent protein loading amounts across all the samples prior to electrophoresis and mass spectrometry (MS) analysis, the protein concentrations were normalized using the Bicinchoninic Acid Assay (BCA) method. The protein aliquots were stored at −80 °C.

### 2.5. SDS-PAGE Electrophoresis

Protein was separated by a 12% SDS-PAGE gel as described by Candiano et al. [27]. First, the gel was fixed for 2 h and stained with Coomassie Brilliant Blue R-250 for 12 h. After staining, the gel was washed with water until the protein bands became clearly visible. Finally, the stained gel was scanned by an Image Scanner (GE Healthcare, IL, USA) at a resolution of 300 dpi.

### 2.6. Protein Digestion and TMT Labeling

The Filter-Aided Sample Preparation (FASP) method [28] was used to perform protein digestion and labeling. About 100 μg of protein extract was mixed with 120 μL reduction buffer (10 mM DTT, 8 M urea, 100 mM TEAB, pH 8.0). The solution was incubated at 60 °C for 1 h. Iodoacetamide (IAA) was added to the solution to achieve a final concentration of 50 mM and reacted in the dark at room temperature for 40 min. Then, the solution was centrifuged at 12,000 rpm for 20 min at 4 °C. The pellet was resuspended in 100 μL of TEAB (100 mM) and centrifuged at 12,000 rpm for 20 min; this step was repeated twice. After washing, 100 μL of TEAB (100 mM) was added, followed by 2 μL of sequencing-grade trypsin (1 μg/μL), and then, the solution was incubated for digestion at 37 °C for 12 h. The digestate was centrifuged at 12,000 rpm for 20 min. Finally, 50 μL of TEAB (100 mM) was added and centrifuged again. The solution was collected and lyophilized using a vacuum refrigerated centrifuge.

For TMT labeling, the lyophilized samples were resuspended in 100 μL of 50 mM triethylammonium bicarbonate (TEAB), and 40 μL aliquots from each sample were taken for labeling. The TMT reagent was equilibrated to room temperature, and 41 μL of anhydrous acetonitrile was added to the TMT reagent. The TMT reagent was vortexed for 5 min to ensure complete dissolution. Then, 41 μL of the TMT labeling reagent was added to each 100 μL sample for mixing, and the mixture was incubated at room temperature for 1 h. Finally, 8 µL of 5% hydroxylamine was added to each sample and incubated for 15 min to quench the labeling reaction. The labeled peptide solutions were lyophilized and stored at −80 °C.

### 2.7. Reverse-Phase Liquid Chromatography (RPLC) Analysis

Reversed-phase separation was performed on an Agilent 1100 Series HPLC System (Agilent Technologies, Santa Clara, CA, USA) with an Agilent Zorbax Extend RP column (5 μm, 150 mm × 2.1 mm). Mobile phase A (2% acetonitrile in ultrapure water) and mobile phase B (98% acetonitrile in ultrapure water) were used. The solvent gradient was as follows: 0–8 min, 98% A; 8.00–8.01 min, 98–95% A; 8.01–38 min, 95–75% A; 38–50 min, 75–60% A; 50–50.01 min, 60–10% A; 50.01–60 min, 10% A; 60–60.01 min, 10–98% A; 60.01–65 min, 98% A. Peptides were separated at a flow rate of 300 μL/min and detected at wavelengths of 210 nm and 280 nm. The eluted peptides were collected from 8 min to 50 min, and the elution fractions were collected at 1 min intervals. The separated peptides were lyophilized and stored at −80 °C for subsequent MS analysis.

### 2.8. MS Analysis

Analyses were performed using a Q Exactive™ Hybrid Quadrupole-Orbitrap mass spectrometer (Thermo Fisher Scientific, Waltham, MA, USA) equipped with a Nanospray Flex source (Thermo, USA). Samples were loaded by a capillary C18 trap column (3 cm × 100 µm) and then separated by a C18 column (15 cm × 75 µm i.d., 2 μm particle size, 100 Å pore size, Acclaim PepMap RSLC) on an EASY-nLC^TM^ 1200 system (Thermo, USA). The flow rate was 300 nL/min, and a 90 min linear gradient from 5% to 100% mobile phase B (0.1% FA in 80% ACN) was applied. Eluent A was 0.1% (*v*/*v*) formic acid (FA) in ultrapure water. The gradient program was as follows: 8% B, 0–55 min; 30% B, 55–79 min; 50% B, 79–80 min; 100% B, 80–90 min; 90–100% B over 5 min.

Full MS scans were acquired in the mass range of 300–1600 m/z with a mass resolution of 70,000, and the automatic gain control (AGC) target value was 1 × 10^6^. The top 10 most intense precursor ions in full MS were fragmented with higher-energy collisional dissociation (HCD) with a collision energy of 30. MS/MS spectra were obtained with a resolution of 17,500, an AGC target of 200,000, and a maximum injection time of 80 ms. The Q-E dynamic exclusion was set for 15.0 s and operated in positive ion mode. Three biological replicates were established for both the PMF treatment group and the control group. Each sample underwent three technical replicates of TMT labeling and MS analysis to assess reproducibility.

### 2.9. Database Search

Proteome Discoverer (version 2.2) was employed to search all of the Q Exactive MS/MS raw data thoroughly against the *L. monocytogenes* protein database. The database searches were conducted with trypsin digestion specificity, and the cysteine alkylation was specified as a fixed modification during the database searching. For protein quantification, TMT 6-plex labeling was selected. The normalization method applied to the TMT data was median normalization. Specifically, this approach standardizes the ratios of all the peptides by referencing the median of the protein ratios and adjusts the median protein ratio to 1 after standardization. A global false discovery rate (FDR) of <0.01 was applied, and peptide groups considered for quantification required at least one peptide per protein group.

### 2.10. Bioinformatic Analysis

Proteins with a fold change >1.2-fold or <0.83-fold and *p*-value < 0.05 were defined as differentially expressed proteins (DEPs). Gene Ontology (GO) analysis was performed using the Blast2GO (Version 2.8.0) tool. Kyoto Encyclopedia of Genes and Genomes (KEGG) pathway enrichment analysis was performed to analyze the biological pathways. GO and KEGG pathway enrichment analysis was performed using a background dataset constructed from the total proteome of *L. monocytogenes* obtained from UniProt. The screening criteria were set as *p*-value < 0.05 and FDR < 0.01.

## 3. Results

### 3.1. Inactivation of L. monocytogenes by PMF

**Figure 1:**
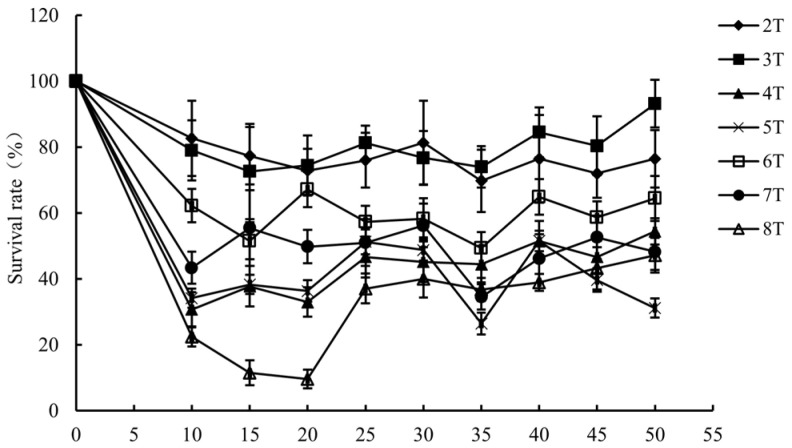
Survival rate of *L. monocytogenes* under PMF treatment.

The inactivation efficiency of *L. monocytogenes* treated by PMF intensities of 2–8 T with pulse numbers of 10–50 is shown in Figure 1. The survival rate decreased after PMF treatment, and the lowest survival fraction was 9.6% when a PMF at the intensity of 8 T with a pulse number of 20 was applied to *L. monocytogenes*. However, the survival rate did not continue to decrease with increasing intensity and pulse number, which may be attributed to the “window effect”, one of the characteristics of electromagnetic fields. Specific microorganisms exhibit biological effects under electromagnetic fields with specific intensity and frequency ranges; thus, the “window effect” includes both the “Frequency (or Time) Window” and the “Intensity (or Power Density) Window” [29]. Due to the influence of the window effect, the sterilization efficacy exhibits a systematic fluctuation trend in response to variations in key parameters such as magnetic field intensity and pulse [30]. Furthermore, series of valley values appear due to “window effect”. The survival rate reached its lowest point at 35 pulses, which was significantly lower than the survival rates at the adjacent pulse numbers of 30 and 40 (*p*-value < 0.05). This finding aligns with the characteristic of “window effect”. Pulses of 35 might be one of the “frequency windows” in the inactivation of *L. monocytogenes* by a PMF at the intensity of 2–8 T. It has been found that different intensities also induce different biological effects. Guo et al. [31] proposed that, owing to the “window effect”, the application of a high-power PMF might not necessarily inhibit microbial growth under specific conditions. Naskar et al. [32] demonstrated that short-duration, low-power PMFs were as effective in inactivating *Enterococcus faecalis* as a prolonged exposure to high-power static fields.

### 3.2. Identification of Differentially Expressed Proteins

**Figure 2:**
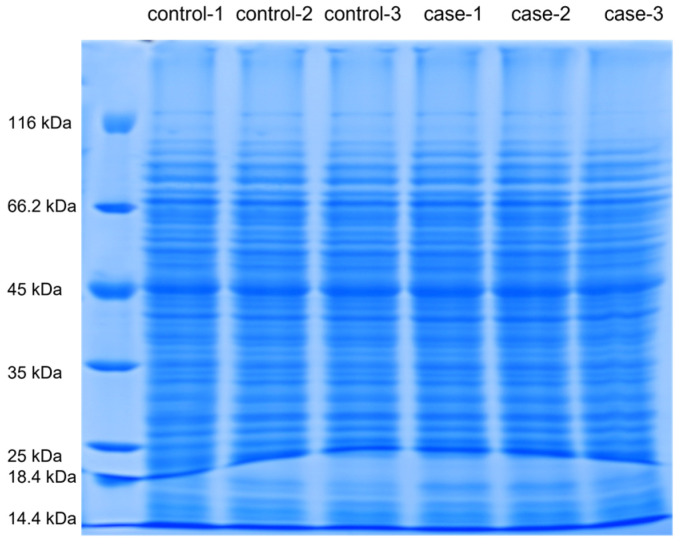
SDS-PAGE gel electrophoresis of proteins extracted from *L. monocytogenes* before and after PMF treatment.

The SDS-PAGE gel electropherogram of proteins from *L. monocytogenes* that were untreated and treated with a PMF (8 T, 20 pulses) is shown in Figure 2. In the SDS-PAGE gel experiments of the control group and the treatment group, 15 μg of protein was precisely loaded into each independent sample well. Overloaded samples can lead to high background noise, bulky bands, streaks, and smears [33]. Therefore, during the experimental procedures, the volume of the sample added should be carefully controlled to maintain the accuracy and reliability of the results. The protein bands were clear and were distributed between 20 and 80 kDa. Moreover, there was a high-abundance band at 50 kDa in each sample. The band was excised from the gel for identification. The complete raw data of the proteomics experiment are provided in the Supplementary Materials. Based on fold change (FC) > 1.2 and *p*-value < 0.05, 101 differentially expressed proteins (DEPs) were screened. Meanwhile, 79 DEPs (65 upregulated and 14 downregulated) were successfully identified, and the protein names and other information are listed in Table 1.

**Figure 3:**
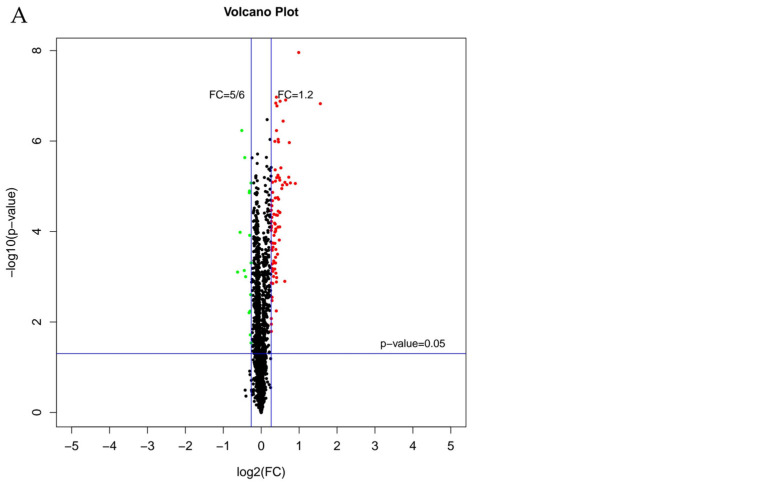

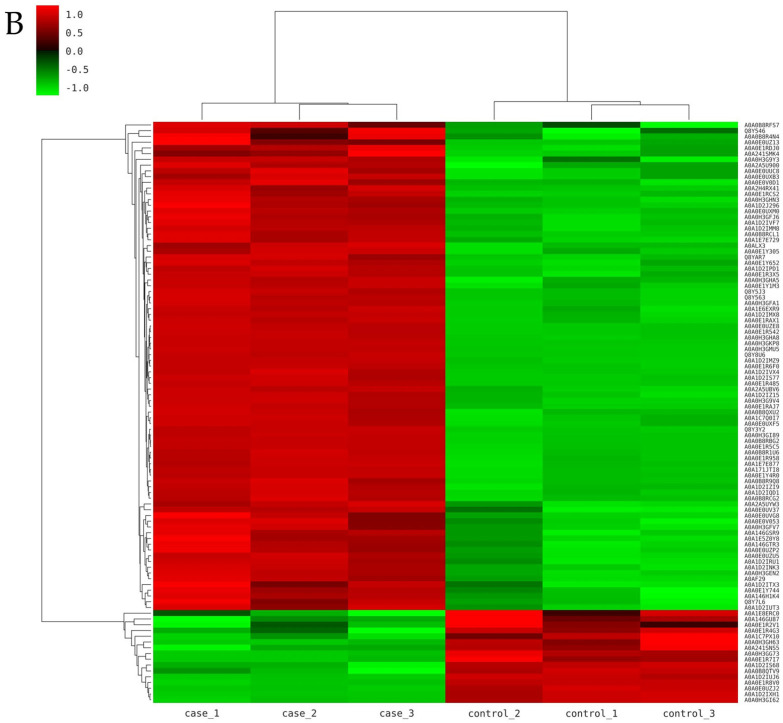
Protein volcano plot (**A**) and clustering heatmap of expression profiles (**B**).

The volcano plot (Figure 3A) showed all DEPs between untreated and treated samples. Black dots in Figure 3A represented proteins that had no significant difference; green dots represented downregulated proteins and red dots represented upregulated proteins. Figure 3B was the clustered heatmap of protein expression patterns. The green lines represented the downregulated proteins, while red lines represented the upregulated proteins. After PMF treatment, most proteins in *L. monocytogenes* were upregulated.

### 3.3. Analysis of Differentially Expressed Proteins

#### 3.3.1. GO Analysis of DEPs

**Figure 4:**
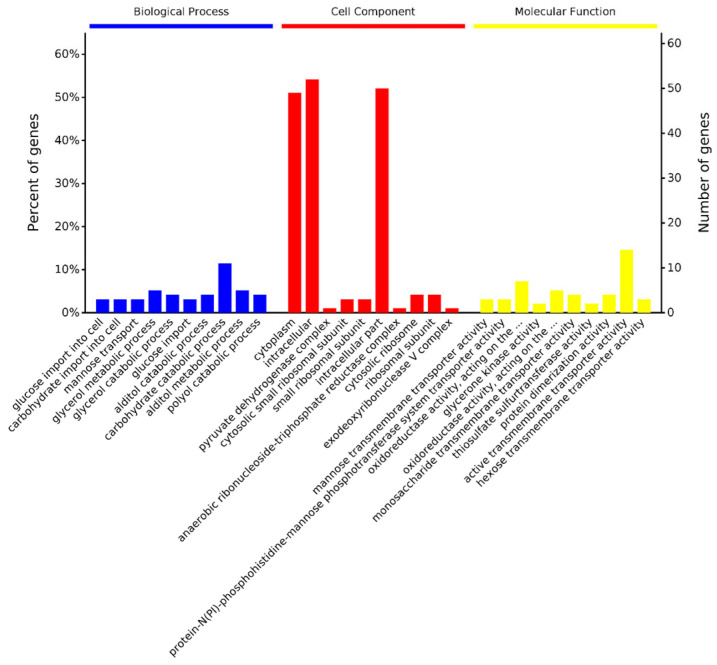
Gene Ontology (GO) functional enrichment analysis.

Bioinformatics analyses were performed to better elucidate the response of *L. monocytogenes* to PMF treatment [34]. GO functional annotation was performed to identify the biological process, cellular components, and molecular functions related to DEPs. The top ten terms sorted by significance in GO analysis are shown in Figure 4. In biological processes, DEPs after PMF treatment were mainly classified into terms such as glucose import into cell, carbohydrate import into cell, and mannose transport. In cellular components, the DEPs were involved in cytoplasm, intracellular regions, pyruvate dehydrogenase complex, and so on. In molecular function, terms in DEPs were dominated by mannose transmembrane transporter activity, protein-N(PI)-phosphohistidine-mannose phosphotransferase system transporter activity, oxidoreductase activity, and so on.

#### 3.3.2. KEGG Analysis of DEPs

**Figure 5:**
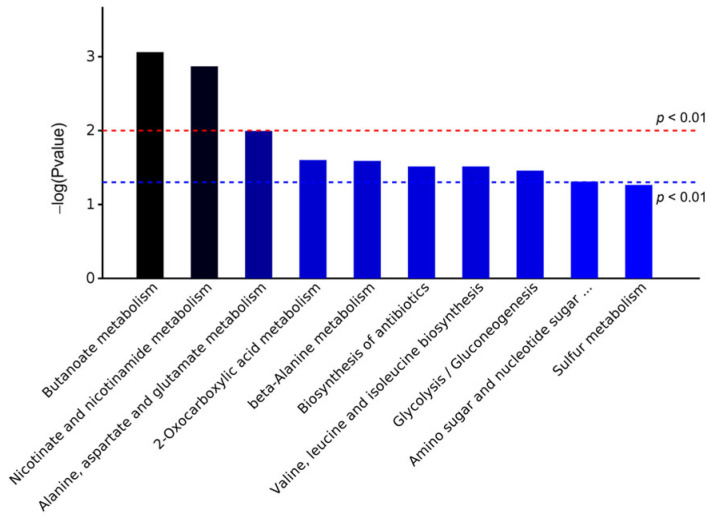
KEGG pathway enrichment analysis.

The KEGG database was used to analyze the pathway enrichment of DEPs. The KEGG database system systematically integrates and stores the functional annotation information of genes and genomes, encompassing a wide range of biological processes [35]. Specifically, it includes core modules such as metabolic pathways, membrane transport mechanisms, signal transduction networks, cell cycle regulation, and conserved sub-pathways across species [36]. This database enables the visualization and functional analysis of cellular biochemical processes, thereby offering substantial support for bioinformatics research. The top 10 pathways of KEGG enrichment for DEPs based on significance are shown in Figure 5. The metabolic pathways involved in DEPs were mainly butanoate metabolism, nicotinate and nicotinamide metabolism, alanine, aspartate and glutamate metabolism, and so on.

Similar to the results of the GO analysis, DEPs participated in metabolic pathways related to glycerol metabolism, sulfur metabolism, and so on. In addition, DEPs participated in metabolic pathways involved in amino acid metabolism.

#### 3.3.3. DEPs Involved in Transportation

PTS mannose/fructose/sorbose transporter subunit IIB (*CDR86_01030*), mannose permease IID component (*manZ_3*), PTS system mannose-specific EIIAB component (*manX_2*), and *Lmo2697* protein (*Lmo2697*) were upregulated after PMF treatment. These proteins belong to the phosphoenolpyruvate-dependent sugar phosphotransferase system (PTS), and the function of the phosphotransferase system is to transport sugars from the environment into bacterial cells [37]. The PTS is a carbohydrate transport and phosphorylation system found in all the different phyla of bacteria and archaea. The PTS not only functions as a carbohydrate transport protein but also regulates many cellular processes by phosphorylating its target proteins or interacting with them in a phosphorylation-dependent manner [38]. Therefore, those proteins participate in biological processes in *L. monocytogenes*, such as glucose intracellular transport, carbohydrate import into the cell, and mannose transport [39]. At the same time, these proteins have certain molecular functions, including mannose transmembrane transporter activity and protein-N(PI)-phosphohistidine-mannose phosphotransferase system transporter activity. The upregulation of these proteins may be attributed to carbon catabolite repression (CCR). The bacteria primarily utilize preferred carbon sources (such as sucrose and fructose) to produce energy. If the preferred carbon source is exhausted, the bacteria will synthesize the enzymes needed to transport those less preferred carbon sources, such as mannose and sorbitol [40,41]. Related studies have shown that bacteria can be susceptible to adaptive evolution depending on substrate availability [42]. After PMF treatment, *L. monocytogenes* might increase the uptake of nutrients (including less preferred carbon sources) from the environment to repair the damage caused by the PMF.

In addition, the cadmium-translocating P-type ATPase (*Lmrg_00327*), which is involved in cation transmembrane transport, was significantly upregulated under the PMF, suggesting enhanced ion transmembrane transport in *L. monocytogenes*. Concurrently, several transporters potentially involved in ion transport were identified, including a putative Zn/Cd/Fe cation exporter (*Fief*), a putative efflux ABC transporter/ATP-binding permease protein (*Lmm7_0961*), and a sodium-dependent phosphate transporter (*LmNIHS28_00558*). The ion transmembrane transport in *L. monocytogenes* might be increased by the PMF. Qian et al. [43] reported that a PMF promoted Ca^2+^ transmembrane transport in *L. monocytogenes*. The acceleration of ion transmembrane transport may lead to the disruption of cell membrane integrity, resulting in the loss of cell viability. However, the SecE protein translocase subunit was downregulated, indicating that intracellular protein transmembrane transport and amino acid transport decreased in *L. monocytogenes*.

#### 3.3.4. DEPs Involved in Transcription and Translation

Concerning transcription, the heat-inducible transcription repressor *HrcA* and the transcriptional regulator *CtsR* were upregulated. *CtsR* participates in the positive regulation of DNA-templated transcription, while *HrcA* participates in the negative regulation of DNA-templated transcription. The transcription of some heat-shock proteins, such as molecular chaperones (e.g., *DnaK* and *GroEL*) and proteases (e.g., *Lon*, *Clp*, *FtsH,* and *DegP*), can be regulated by *HrcA* [44]. Therefore, the upregulation of *HrcA* indicates that the heat shock response was triggered to enable cells to resist the PMF to a certain extent.

The DEPs involved in the translation process were 30S ribosomal protein S14 type Z (*rpsN*) and 50S ribosomal protein L35 (*rpmI*). The downregulation of these two proteins might not only indicate a decreased tolerance of *L. monocytogenes* to the PMF but also suggest that PMF potentially affects ribosomal function, thereby disrupting the translation processes. Zheng et al. [45] reported that the abundance of 30S ribosomal proteins S14 and S18, as well as 50S ribosomal proteins L13, L18, and L20, was reduced in the ΔclpP mutant strain, and the reduction might contribute to the decreased tolerance of the ΔclpP mutant strain to linezolid or minocycline. Liu et al. [46] also demonstrated that high hydrostatic pressure (HHP) could upregulate ribosome-related pathways, including the expression of 30S and 50S ribosomal proteins, and HHP might disrupt ribosomal function, consequently impairing transcription and translation processes and ultimately contributing to the mortality of *L. monocytogenes*. Tian et al. [47] found that both high-voltage short-time ohmic (HVST) and low-voltage long-time ohmic (LVLT) caused greater damage to ribosomal proteins, stalling the protein translation process.

#### 3.3.5. DEPs Involved in Carbohydrate Metabolism

Carbohydrates play a pivotal role in bacterial physiology not only supplying the essential energy required for cellular life processes but also acting as a critical carbon source for the synthesis of various biomolecules [48]. The DEPs involved in carbohydrate metabolic processes include PTS-dependent dihydroxyacetone kinase, the dihydroxyacetone-binding subunit dhaK (*dhaK_2*), fructosamine deglycase (*Lmm7_2086*), a putative dihydroxyacetone kinase, a C-terminal domain protein (*dhaL*), 2,3-bisphosphoglycerate-dependent phosphoglycerate mutase (*gpmA*), Lmo2697 protein (*Lmo2697*), and tagatose 1,6-diphosphate aldolase (*lacD*). Notably, all these proteins were significantly upregulated following PMF treatment.

Among them, *dhaK_2* and *dhaL* participate in glycerol metabolism; *gpmA* is related to glycolysis; and *lacD* is associated with lactose catabolism via the tagatose-6-phosphate pathway. Dihydroxyacetone (dha) kinases are homologous proteins that use different phosphoryl donors, and they are also multiphosphorylation proteins of the phosphoenolpyruvate-dependent carbohydrate-phosphotransferase system in bacteria [49]. Dha kinase consists of *dhaL*, *dhaK,* and the multiphosphorylation protein DhaM (*dhaM*) [50]. The upregulation of *dhaK* may provide substrates for the production of different types of carbohydrates [51]. Upregulation of *dhaL* and *dhaM* was also observed in the presence of methanol. *GpmA* is a key enzyme involved in glycolysis [52], converting 2- phosphoglycerate to 3-phosphoglycerate in glycolysis. The upregulation of *gpmA* indicates the increase in glycolysis of *L. monocytogenes* treated with a PMF. *GpmA* is also upregulated when Bacillus anthracis is exposed to hydrogen peroxide [53]. *LacD* transforms tagatose-1, 6-diphosphate to glyceraldehyde-3-phosphate (G3P) and dihydroxyacetone phosphate (DHAP) [54], which are metabolized via the glycolysis pathway. The upregulation of *lacD* might result in the acceleration of glycolysis pathway. *L. monocytogenes* might accelerate carbohydrate metabolism to respond to a PMF.

#### 3.3.6. DEPs Involved in Amino Acid Metabolism

Amino acids serve as the fundamental building blocks for the synthesis of enzymes and proteins in bacteria. Their concentrations not only influence bacterial growth but also play an essential role in cell wall and membrane biosynthesis, material transport, and biofilm formation [55]. Environmental disturbances cause changes in the amino acid metabolism of microorganisms [56]. The DEPs involved in amino acid metabolism were glutamate decarboxylase (*LMRG_01479*), glutamate decarboxylase (*gadG*), ketol-acid reductoisomerase (NADP(+)) (*ilvC*), and cystathionine beta-lyase (*metC*). They were all upregulated as were DEPs involved in carbohydrate metabolism.

*LMRG_01479* and *gadG* are associated with glutamate metabolic processes, while *ilvC* is related to leucine, isoleucine, and valine biosynthetic processes. Glutamate decarboxylase catalyzes the irreversible conversion of L-glutamate to γ-aminobutyric acid (GABA) [57]. An incremental expression of the glutamate decarboxylase system explains one of the mechanisms underlying acid resistance in microorganisms [58]. The metabolic processes in *L. monocytogenes* that alleviate acid stress primarily involve proton-consuming reactions, including glutamate decarboxylation, arginine/agmatine deimination, and fermentative acetoin production [59]. Under PMF conditions, the *gadG* gene, which is associated with acid tolerance, was found to be upregulated by 1.43-fold compared with the control group. This gene maintains intracellular pH homeostasis through the GABA metabolic pathway, thereby enhancing the cell’s adaptability and survival in acidic environments. Boura et al. [60] indicated that glutamate decarboxylase plays a crucial role in the acid tolerance and oxidative stress resistance of *L. monocytogenes*. Branched-chain amino acid (BCAA) biosynthesis starts with pyruvate and threonine, and *ilvC* participates in the second or third step of the biosynthesis of *Val*, *Leu*, and *Ile*, respectively. Moreover, in the Ehrlich pathway, *ilvC* utilizes nicotinamide adenine dinucleotide phosphate (NADPH) as a cofactor. *IlvC* plays some roles in managing stress, such as pH and starvation [61]. It is reported that the downregulation of *ilvC* affected the ability of Mycobacterium tuberculosis to persist and its survival in macrophages and in mice [62]. In bacteria, cystathionine beta-lyase (*metC*) is responsible for the hydrolysis of L-cystathionine (*L-Cth*) to L-homocysteine (*L-Hcys*), pyruvate, and ammonia [63]. *MetC* not only participates in transsulfuration in amino acid metabolic process but also participates in transsulfuration in sulfur metabolic process. The upregulation of *metC* may also enhance the sulfur metabolism. The enhancement of the amino acid metabolism might improve the survival ability of *L. monocytogenes* exposed to a PMF.

#### 3.3.7. DEPs Involved in Nicotinate and Nicotinamide Metabolism

As a core component in the metabolic pathways of nicotinic acid and nicotinamide, niacinamide exerts anti-oxidative stress and anti-inflammatory effects through the regulation of cellular energy metabolism [64]. The DEPs including fumarate reductase (*LmNIHS28_02228*), cystathionine beta-lyase (*metC*), *Lmo0047*, *Lmo2213*, and putative nico-tinamidase (*pncA*) were related to nicotinate and nicotinamide metabolism. Nicotinamidase is capable of catalyzing the conversion of nicotinamide into nicotinic acid. Feng et al. [45] demonstrated that *pncA* plays a critical role in the salvage synthesis pathway of nicotinamide adenine dinucleotide (NAD+). The PMF affected the homeostasis of *L. monocytogenes*, and *pncA* was upregulated to help *L. monocytogenes* to maintain metabolic homeostasis and cope with PMF-induced damage. Shats et al. [65] demonstrated that the *pncA* gene in *Escherichia coli* plays a critical role in responding to adverse environmental stimuli by modulating the nicotinamide metabolic pathway, thereby maintaining intracellular metabolic homeostasis effectively. In general, the nicotinate and nicotinamide metabolism was increased to help *L. monocytogenes* survive in a PMF.

#### 3.3.8. DEPs Involved in Other Metabolism

Aldehyde–alcohol dehydrogenase (*LMRG_01332*) was upregulated after PMF treatment and participates in the butanoate metabolism, biosynthesis of antibiotics, glycolysis/gluconeogenesis, and biosynthesis of secondary metabolites in *L. monocytogenes*. Aldehyde–alcohol dehydrogenases (ADHEs) convert acyl-CoAs and aldehydes to their corresponding alcohols and play an important role in butanol biosynthesis. Martin et al. [66] identified the aldehyde–alcohol dehydrogenase as the primary bottleneck in butanol production, and increasing the bi-functional aldehyde–alcohol dehydrogenase resulted in a much larger improvement in the butanol titer than increasing any other butanol pathway enzymes. Furthermore, Chlamydomonas reinhardtii with the upregulation of aldehyde–alcohol dehydrogenase increased its survival under dark anoxia [67]. Marina Uroz et al. [68] assert that cell cycle progression and communication between cellular components are mediated by two-component signal transduction systems and signaling pathways involving the activation of transcription factors. In addition, bacterial cells can adapt their transcriptional cues to changing environments and respond correctly to stimulation by transcriptional regulators [69]. The upregulation of aldehyde–alcohol dehydrogenase may be a self-protective stress response of *L. monocytogenes*, which increases its survival ability under PMF treatment.

## 4. Conclusions

A proteomic analysis of *L. monocytogenes* exposed to a PMF was performed to investigate the relationship between changes in protein expression and loss of cell viability. Under the PMF treatment at 8 T with 20 pulses, 101 differentially expressed proteins (DEPs) were annotated, and 79 proteins were successfully identified. Meanwhile, 65 proteins were upregulated, and 14 proteins were downregulated. These DEPs were related to transport, cytoplasmic processes, metabolism, and other functions of *L. monocytogenes*. The PMF affected the transport capacity and ribosomal structure in *L. monocytogenes*. *L. monocytogenes* transiently enhanced nutrient uptake; accelerated the metabolism of carbohydrates, amino acids, nicotinate, and nicotinamide; and activated the heat-shock response as compensatory adaptations to the PMF. These findings indicate that, although a PMF disrupts essential biological functions and metabolic pathways, eventually resulting in cell death, *L. monocytogenes* can temporarily alleviate PMF-induced stress through these adaptive responses. However, the adaptive mechanisms were insufficient to fully counteract the cumulative damage, ultimately leading to impaired cellular viability. Future research should employ integrated multi-omics approaches, combining proteomics, transcriptomics and metabolomics, to achieve a more in-depth and comprehensive elucidation of the molecular mechanisms underlying the response of *L. monocytogenes* to a PMF.

## Figures and Tables

**Table 1:** DEPs identified by MALDI-TOF MS.

Spot No.	Gene/ORF Name	Description	Accession No.	pI/MW (kDa)	Scores	Seq Cov (%)	Fold Change
1	*ylbF* *	Regulatory protein ylbF	A0A0E1R7I7	5.68/18.7	5.81	6	−1.54
2	*LMRG_00836*	UPF0176 protein LMRG_00836	A0A0H3GG73	4.98/36.3	219.75	59	−1.47
3	*rpmI*	50S ribosomal protein L35	A0A0E1R8V0	12.56/7.7	116.31	35	−1.43
4	*hemA* *	Glutamyl-tRNA reductase	A0A0H3GH63	5.33/49.2	21.34	5	−1.37
5	*CDR86_15435*	tRNA-dihydrouridine synthase	A0A1D2IUJ6	6.44/36.8	326.28	64	−1.35
6	*A410_1127* *	UPF0358 protein A410_1127	A0A241SNS5	8.63/12.6	2.5	10	−1.33
7	*SAMD00023519_01241*	Dipicolinate synthase	A0A146GU87	8.66/20.8	17.66	13	−1.25
8	*lysP* *	Lysine-specific permease	A0A0E1R4G3	9.41/53.1	4.52	2	−1.23
9	*CDR86_13105*	Carbonic anhydrase	A0A1D2IXH1	4.97/27.2	405.4	49	−1.23
10	*BB718_05970*	DNA-binding response regulator	A0A1D2IS68	6.13/28.5	61.22	22	−1.23
11	*secE* *	Protein translocase subunit SecE	A0A0E1R2V1	9.54/6.9	2.24	14	−1.20
12	*LmNIHS28_00558* *	Sodium-dependent phosphate transporter	A0A0B8QTV9	5.45/59.7	5.77	1	−1.20
13	*rpsN*	30S ribosomal protein S14 type Z	A0A0E0UZJ2	10.49/7.1	21.41	34	−1.20
14	*CDR86_09705*	Rhodanese-like domain-containing protein	A0A1C7PX10	4.84/10.8	52.88	58	−1.20
15	*LMRG_01479*	Glutamate decarboxylase	A0A0H3GMU5	5.22/53.5	305.63	38	2.95
16	*LMRG_01332*	Aldehyde–alcohol dehydrogenase	A0A0H3GKP8	6.93/94.6	2625.08	57	1.99
17	*LMRG_01480* *	Glutamate/gamma-aminobutyrate antiporter	A0A0H3GFA1	9.09/55.1	21.75	4	1.87
18	*lmo2067*	Lmo2067 protein	Q8Y5J3	5.15/36.8	38.58	19	1.71
19	*inlB* *	Internalin B	A0A0E1R485	9.41/71.2	20.55	5	1.67
20	*CDR86_05685*	GNAT family N-acetyltransferase	A0A1D2IS77	4.94/10.2	59.52	23	1.65
21	*LMRG_00327*	Cadmium-translocating P-type ATPase	A0A0H3G9V4	5.72/67.6	238.61	23	1.60
22	*spsB*	Signal peptidase I	A0A0E1R6F0	5.19/21.1	50.79	28	1.56
23	*LmNIHS28_02228*	Fumarate reductase	A0A0B8RBG2	5.94/54.5	754.17	57	1.49
24	*dhaK_2*	PTS-dependent dihydroxyacetone kinase, dihydroxyacetone-binding subunit dhaK	A0A0E1R958	4.93/34.9	123.73	19	1.48
25	*gadG*	Glutamate decarboxylase	A0A0E1RAJ7	5.22/54.8	76.2	15	1.43
26	*lmo0796*	Lmo0796 protein	Q8Y8U6	4.84/19.3	404.6	88	1.41
27	*LMM7_2086* *	Fructosamine deglycase	A0A0E0UXM0	5.54/38	21.25	9	1.41
28	*NT04LM_1576*	Lipoprotein, putative (Fragment)	A0A0E1Y652	4.77/8.4	23.34	19	1.40
29	*CDR86_01030*	PTS mannose/fructose/sorbose transporter subunit IIB	A0A1C7Q0I7	6.34/17.2	21.69	17	1.40
30	*CXL08_12865*	NAD-dependent dehydratase	A0A1D2IMX8	6.38/22.7	76.14	35	1.39
31	*LMM7_2092* *	Putative transcription regulator, GntR family	A0A0E0UXB3	5.45/28.0	2.09	3	1.39
32	*dhaL*	Dihydroxyacetone kinase, C-terminal domain protein	A0A0E0UZE8	5.33/21.5	228.46	52	1.37
33	*CDR86_02755*	Carbohydrate kinase	A0A1D2IMM8	4.96/40.6	35.57	10	1.37
34	*lwe2587*	lipoprotein	A0ALX3	5.91/32.9	1369.32	53	1.36
35	*gpmA*	2,3-Bisphosphoglycerate-dependent phosphoglycerate mutase	A0A0H3GI89	5.69/26.4	285.71	39	1.36
36	*manZ_3* *	Mannose permease IID component	A0A0E1RAX1	8.34/31.8	35.5	7	1.36
37	*CDR86_08010*	Anaerobic ribonucleoside-triphosphate reductase	A0A1D2IZ15	5.94/82.1	131.03	24	1.35
38	*BN389_17220*	Epimerase family protein SE_0553	A0A0E1RDJ0	7.68/34.6	20.27	17	1.35
39	*NT04LM_1565*	DNA protection during starvation protein 2	A0A0E1Y4R0	4.93/18.0	915.53	79	1.33
40	*LMRG_00977*	Short chain dehydrogenase	A0A0H3GHA8	6.19/20.9	91.47	38	1.33
41	*LMRG_00977*	Lmo1261 protein	Q8Y7L6	9.41/42.3	122.24	18	1.33
42	*manX_2*	PTS system mannose-specific EIIAB component	A0A0E1R542	9.33/19.7	262.23	46	1.32
43	*lmo2697*	Lmo2697 protein	Q8Y3Y2	4.7/13.4	115.1	54	1.32
44	*Fief* *	Putative Zn/Cd/Fe cation exporter	A0A0E0UZ13	5.78/31.8	10.55	6	1.32
45	*CLN77_13670*	FMN-binding protein	A0A2H4RX41	6.65/32.7	1370.82	50	1.31
46	*psuG*	Pseudouridine-5’-phosphate glycosidase	A0A1D2IMZ9	4.84/32.5	385.23	61	1.31
47	*SAMD00023519_01958* *	Putative activator of (R)-hydroxyglutaryl-CoA	A0A146GTR3	6.34/164.1	61	7	1.31
48	*A4P56_12940* *	Cell surface protein	A0A2A5UYW3	5.15/34.6	14.81	16	1.31
49	*AF973_12935* *	Amino acid ABC transporter permease	A0A1E6EXR9	9.66/23.9	9.96	6	1.31
50	*CDR86_13550*	Pyruvate oxidase	A0A1D2INK3	5.02/62.8	266.31	35	1.30
51	*lmo2213*	Lmo2213 protein	Q8Y563	7.21/19.5	101.16	35	1.30
52	*SAMD00023519_01961* *	PTS mannose transporter subunit IIC	A0A146GSR9	5.54/32.2	24.98	4	1.30
53	*AJL15_04430*	FMN-binding protein	A0A1E7E877	6.65/32.7	1328.39	50	1.29
54	*pdxH*	Putative general stress protein 26 putative pyridoxamine/pyridoxine 5’-phosphate oxidase	A0A0E0UZU5	4.68/15.7	59.03	35	1.29
55	*lacD*	Tagatose 1,6-diphosphate aldolase	A0A0E1R5C5	5.1/37.9	599.72	68	1.28
56	*hrcA*	Heat-inducible transcription repressor HrcA	A0A0E1Y305	5.58/40.4	67.28	18	1.28
57	*AJZ74_10515* *	Serine/threonine protein phosphatase	A0A1E5Z0Y8	5.08/26.7	16	14	1.28
58	*SAMD00023520_02065* *	Cyclic nucleotide-binding protein	A0A146H1K4	9.22/32.8	3.94	6	1.27
59	*deoC*	Deoxyribose-phosphate aldolase	A0A1D2IZI9	5.39/23.5	454.21	70	1.26
60	*mgtA* *	Magnesium-translocating P-type ATPase	A0A1D2IRU1	7.99/94.8	52.56	9	1.26
61	*ilvC*	Ketol-acid reductoisomerase (NADP(+))	A0A0H3GHN3	5.36/36.4	33.9	16	1.26
62	*LMRG_02097*	ATP-dependent Clp protease ATP-binding subunit ClpE	A0A0H3GFJ6	5.21/80.2	226.18	31	1.25
63	*LMM7_0960*	Putative efflux ABC transporter, ATP-binding protein (N-terminal part)	A0A0E0UVG8	6.11/25.9	41.68	30	1.25
64	*LMM7_0961* *	Putative efflux ABC transporter, ATP binding and permease protein	A0A0E0UUC8	7.18/41.3	15.71	5	1.24
65	*pncA* *	Putative nicotinamidase	A0A0E0V053	4.77/23.5	5.27	3	1.24
66	*CDR86_04330*	Carnitine transport ATP-binding protein OpuCA	A0A1D2IQD1	5.16/45.2	289.6	52	1.23
67	*gabD*	NAD-dependent succinate-semialdehyde dehydrogenase	A0A2A5UBV6	6.09/53.1	335.88	38	1.23
68	*metC* *	Cystathionine beta-lyase	A0A0E0UXF5	5.41/41.7	4.85	2	1.23
69	*mntA*	Manganese-binding lipoprotein MntA	A0A2A5U900	5.52/34.4	717.31	46	1.22
70	*ctsR*	Transcriptional regulator CtsR	A0AF29	6.23/17.5	90.67	37	1.22
71	*LmNIHS28_01175*	UPF0473 protein LmNIHS28_01175	A0A0B8QXU2	3.89/12.1	59.49	29	1.22
72	*phoU* *	Phosphate-specific transport system accessory protein PhoU	A0A1D2ITX3	5.05/25.0	7.64	13	1.22
73	*lmo0047*	Lmo0047 protein	Q8YAR7	4.56/22.7	126.33	35	1.21
74	*BB664_03185*	Lipase	A0A1D2J296	4.61/40.4	40.15	14	1.21
75	*CDR86_03830*	DUF1049 domain-containing protein	A0A1D2IVF7	9.33/12.7	29.94	14	1.21
76	*uspF* *	Putative universal stress protein UspA and related nucleotide-binding protein	A0A0E0V0D1	8.88/17.2	11.51	8	1.21
77	*opuCA*	Glycine betaine/carnitine/choline transport ATP-binding protein OpuCA	A0A0E1RCS2	5.29/36.7	66.89	34	1.20
78	*lmo2230* *	Lmo2230 protein	Q8Y546	4.87/15.9	7.32	19	1.20
79	*Ung* *	Uracil-DNA glycosylase	A0A0B8RFS7	7.91/26.4	4.41	4	1.20

* Proteins with a score below 20 or coverage lower than 10% are categorized as low confidence.

## Data Availability

The original contributions presented in this study are included in the article/Supplementary Materials. Further inquiries can be directed to the corresponding author.

## Supplementary Materials

The following supporting information can be downloaded at: https://www.mdpi.com/article/10.3390/foods14111871/s1, Table S1: Raw data of proteomic experiments.

## Abbreviations

Abbreviation	Term
PMF	Pulsed magnetic field
*L. monocytogenes*	*Listeria monocytogenes*
GO	Gene Ontology
KEGG	Kyoto Encyclopedia of Genes and Genomes
HPP	High-Pressure Processing
PEF	Pulsed electric field
TMT	Tandem Mass Tag
BHI	Brain heart infusion broth
CFU	Colony-forming unit
IAA	Iodoacetamide
TEAB	Triethylammonium bicarbonate
MS	Mass spectrometry
FA	Formic acid
AGC	Automatic gain control
HCD	Higher-energy collisional dissociation
FDR	False discovery rate
DEPs	Differentially expressed proteins
FC	Fold change
CCR	Carbon catabolite repression
HVST	High-voltage short-time ohmic
LVLT	Low-voltage long-time ohmic
ADHE	Aldehyde–alcohol dehydrogenases
T	Tesla
HHP	High hydrostatic pressure
